# Supplementary material for: Analysis of PPARγ Signaling Activity in Psoriasis
Source: Int J Mol Sci. 2021 Aug 10;22(16):8603. doi: 10.3390/ijms22168603 (PMC8395241; doi:10.3390/ijms22168603)
Supplement: Supplementary file 1 [file ijms-22-08603-s001.zip › Supplemental materials_Analysis of PPARg signaling activity in psoriasis/Pathway models/Models images and html files/PPARG inhibits phenotypes assotiated with psoriasis/245535.html]

autoimmunity


# Disease autoimmunity

|  |  |
| --- | --- |
| URN | urn:agi-meshdis:Autoimmunity |
| Total Entities | 0 |
| Connectivity | 3949 |
| Name | autoimmunity |

---

|  |  |
| --- | --- |
| Pathway | PPARG inhibits phenotypes assotiated with psoriasis |
|  | Neighbors of autoimmunity |
|  | 2\_3\_Effects of thyroid hormone deficiency: levothyroxine systemic effects |

---

|  |  |
| --- | --- |
| MedScan ID | 9023919 |

---

|  |  |
| --- | --- |
| Alias | auto sensitizations |
|  | autoallergy |
|  | auto immunization |
|  | Self Recognition (Immune) |
|  | autoimmunization |
|  | autoimmunology |
|  | autosensitisations |
|  | autoimmunizations |
|  | auto-immunity |
|  | autoallergies |
|  | auto-immunology |
|  | autosensitisation |
|  | Autoimmune Status |
|  | auto sensitization |
|  | Autoimmune States |
|  | autoimmunisations |
|  | auto immunizations |
|  | Autoimmunities |
|  | autosensitization |
|  | autoimmunisation |
|  | Autoimmune State |
|  | Autoimmunity |
|  | auto immunisations |
|  | auto sensitisation |
|  | auto sensitisations |
|  | auto immunisation |

---

|  |  |
| --- | --- |
| MeSH ID | D015551 |
|  | M0023919 |

---

|  |  |
| --- | --- |
| MeSH Heading | Autoimmunity |

---
